# A Rare Case of ST-elevation Myocardial Infarction After Blunt Chest Trauma

**DOI:** 10.7759/cureus.7710

**Published:** 2020-04-17

**Authors:** Oscar Perez, Raunak M Nair, Tariq Kewan, Mohammed J Al-Jaghbeer

**Affiliations:** 1 Internal Medicine, Cleveland Clinic - Fairview Hospital, Cleveland, USA; 2 Pulmonary and Critical Care, Cleveland Clinic, Cleveland, USA

**Keywords:** st-elevation myocardial infarction (stemi), chest pain, blunt chest trauma, balloon angioplasty with stent

## Abstract

Myocardial infarction (MI) after blunt chest trauma (BCT) is a rare but potentially life-threatening situation that should be addressed immediately in a patient who presents to the ED. Early management is directly related to favorable outcomes. Here we describe a case of ST-elevation MI after BCT.

## Introduction

Myocardial Infarction (MI) or simply termed as ‘heart attack’, is one of the most common causes of death in the United States. Approximately every 40 s, an American will have an MI [1]. The estimated annual incidence of MI is 605 000 new attacks and 200 000 recurrent attacks [1-2]. In young patients, the incidence is significantly lower [2]. Smoking, a family history of coronary artery disease (CAD), and male sex appear to be the most important risk factors associated with developing a heart attack in this population [3]. Though the most common underlying pathophysiology is related to atherosclerosis and plaque disruption, any obstruction to the blood flow in the coronary arteries could potentially lead to an MI [4-5]

The ST-elevation myocardial infarction (STEMI) due to coronary artery damage is a rare but potential complication of blunt chest trauma (BCT) [6-7]. Multiple case reports have described damage to the left anterior descending artery (LAD), which may be due to its proximity to the chest wall [8]. The shear force applied to the coronary arteries during such trauma can lead to intimal tear and intraluminal thrombosis. Vascular rupture, embolism to the coronary arteries, fissuring of an atherosclerotic plaque with dislodgment of plaque material, and vascular spasm at the site of the injury are also potential triggers [9]. We report the case of a STEMI in a 37-year-old male after a BCT.

## Case presentation

A 37-year-old male patient with a medical history significant for intellectual disability presented to the ED after being assaulted. The patient had suffered a severe blunt injury to his head, face, and chest. He was subjected to multiple physical assaults including several punches and kicks in his face and chest. Though he did not lose consciousness, following the incident he started to experience substernal crushing chest pain. The pain was 2/10 initially but began to progressively increase in intensity. He described it as a pressure-like sensation and denied any radiation to his left arm, jaw, or neck. The pain was associated with diaphoresis, but he denied having nausea, vomiting, shortness of breath, or palpitations.

**Figure 1:**
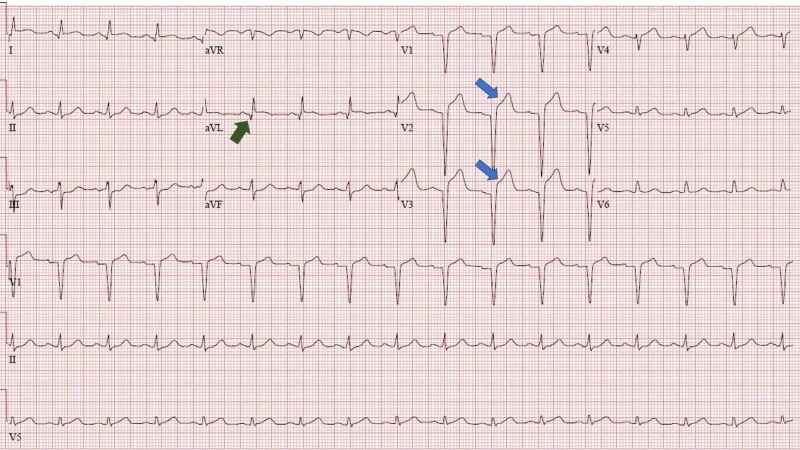
EKG at admission showing ST-segment elevation in V2, V3, and aVL. Q waves in aVL also present. EKG, electrocardiogram

On arrival to ED, his vital signs were stable. On physical examination, he had a bruise on the left side of his chest with bluish discoloration over the site of the injury. He also had multiple bruises over his face, lower extremities, and buttocks. CT scan of the brain and cervical spine did not reveal any abnormalities. His chest pain worsened in the ED to 10/10. An electrocardiogram (EKG) was performed, and it showed normal sinus rhythm with ST-segment elevation in V2, V3, and aVL along with Q waves in aVL (Figure 1). Troponin T: 0.487 mg/dL.

**Figure 2:**
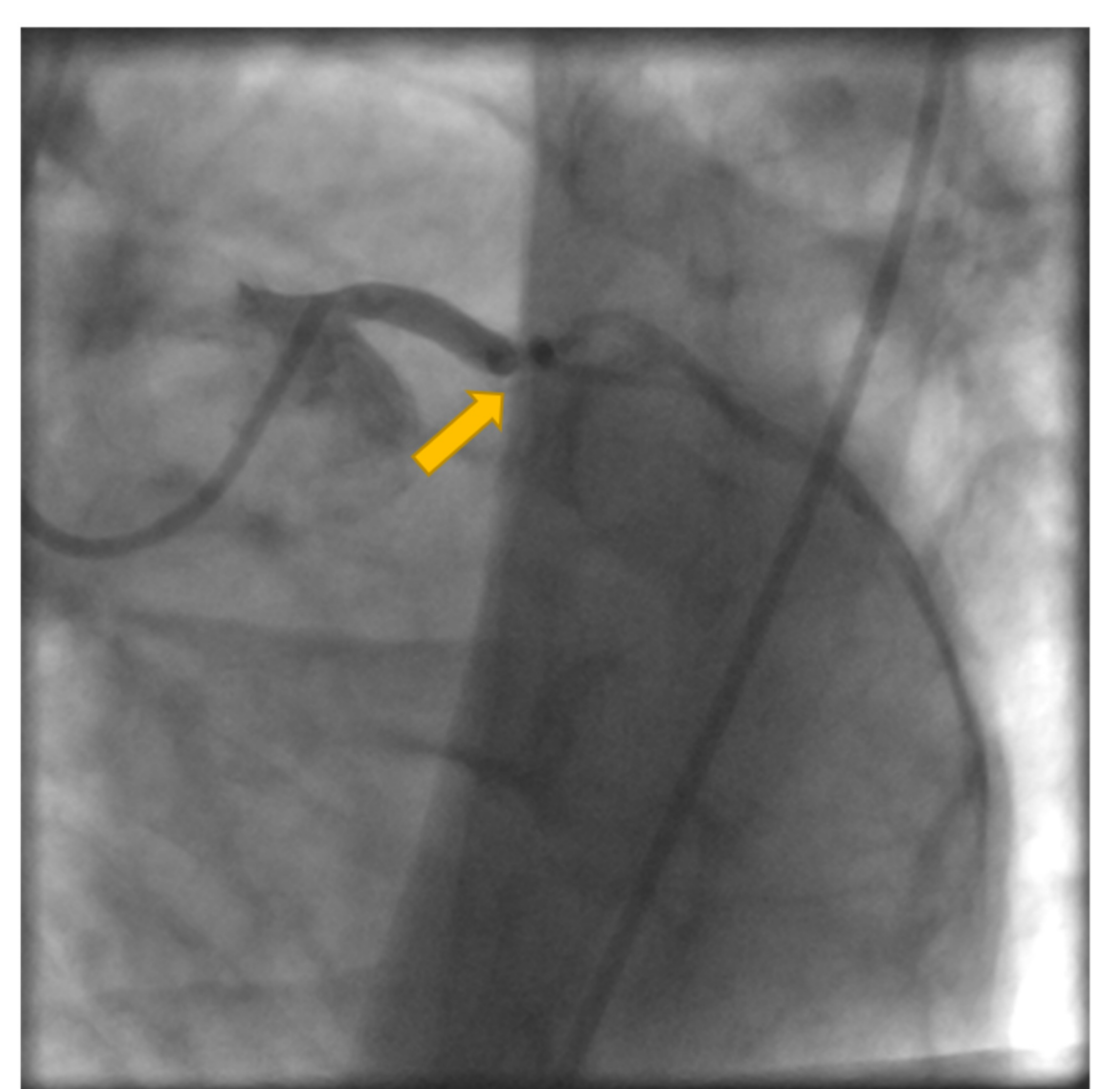
Coronary angiogram showing total occlusion of the LAD. LAD, left anterior descending artery

**Figure 3:**
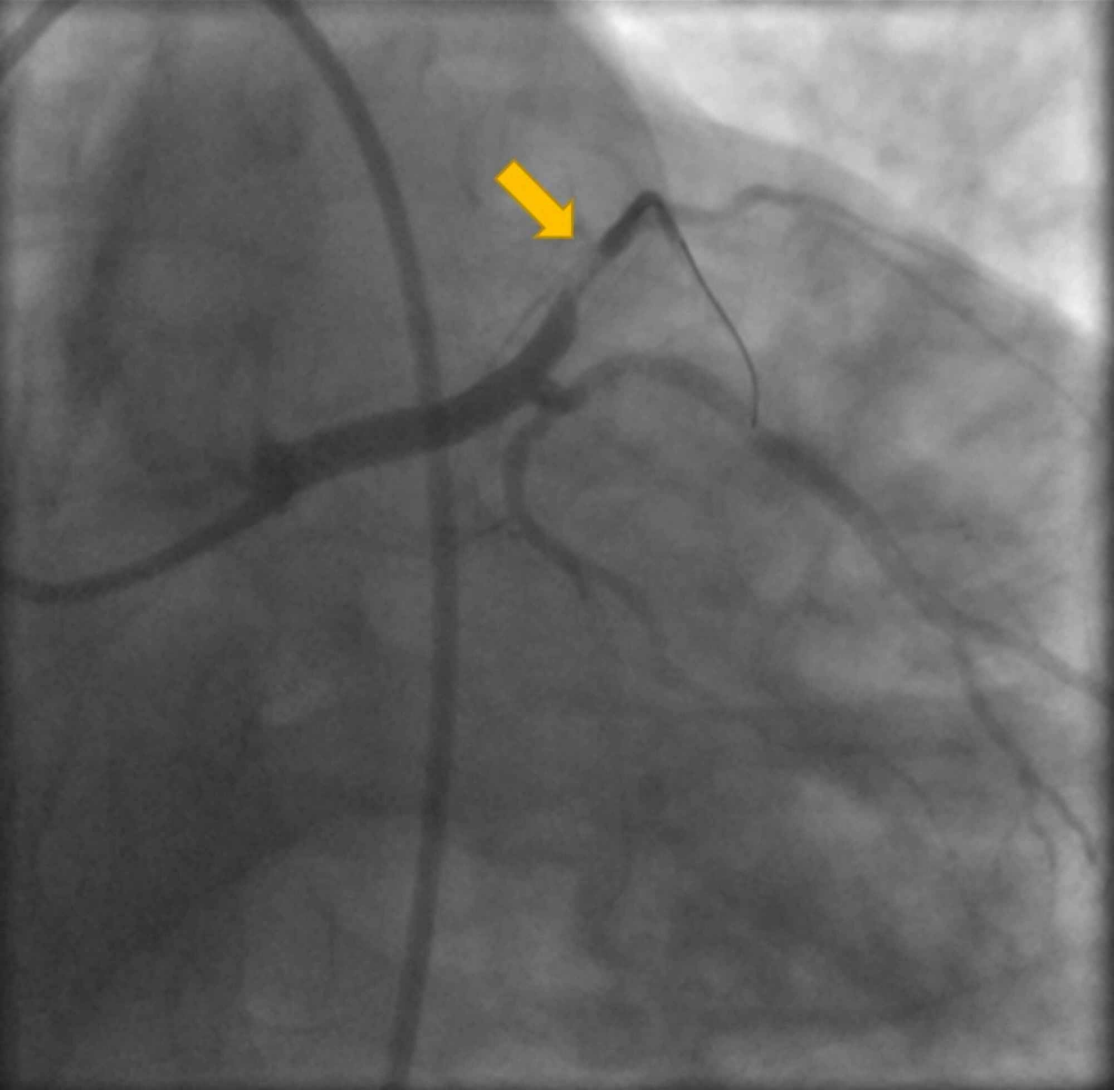
Percutaneous transluminal coronary angioplasty performed.

**Figure 4:**
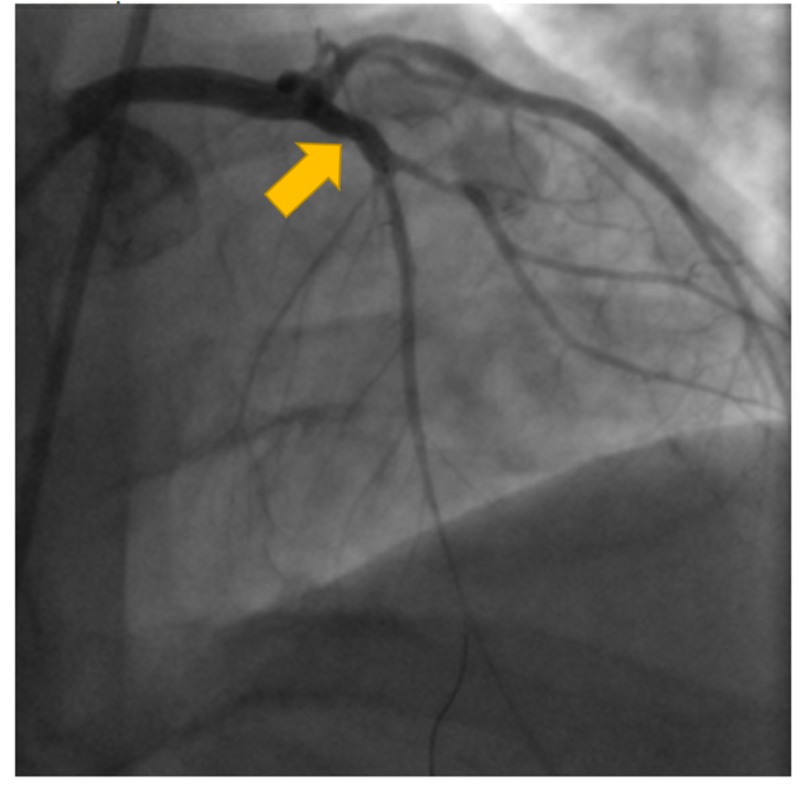
Stent placed successfully with flow to LAD. LAD, left anterior descending artery

**Figure 5:**
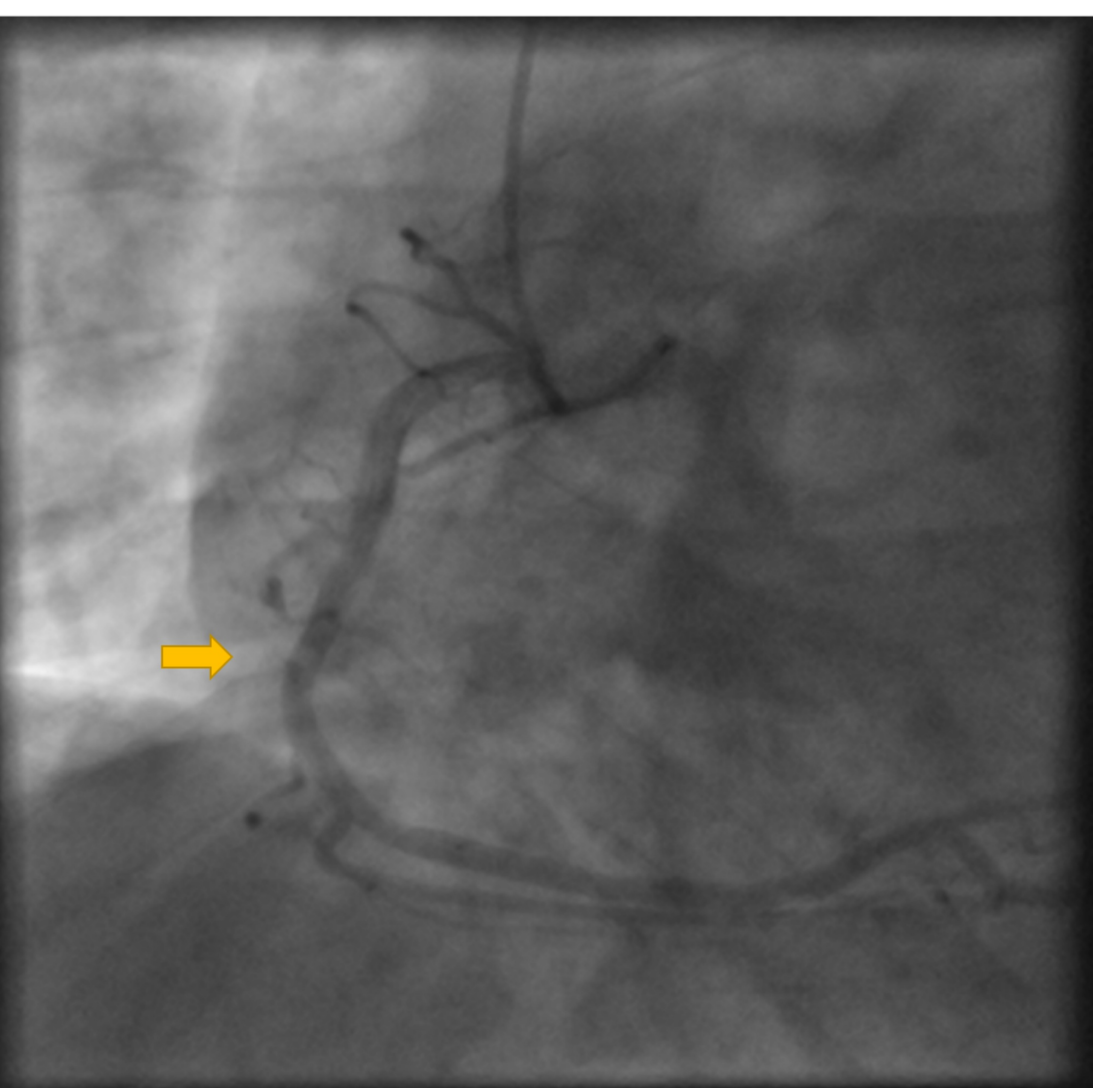
RCA without any coronary artery disease. RCA, right coronary artery

He was given 325 mg aspirin, atorvastatin, and metoprolol. The patient was urgently transferred to the catheterization lab for possible percutaneous revascularization. Coronary angiogram revealed acute total obstruction of the LAD (Figure 2). Angioplasty with stenting of a single drug-eluting stent was performed in proximal LAD (Figures 3-4). Other vessels did not show stenosis (Figure 5). Given the characteristic angiographic findings, other studies such as intravascularultrasound (IVUS) or optical coherence tomography (OTC) was not required. 

**Figure 6:**
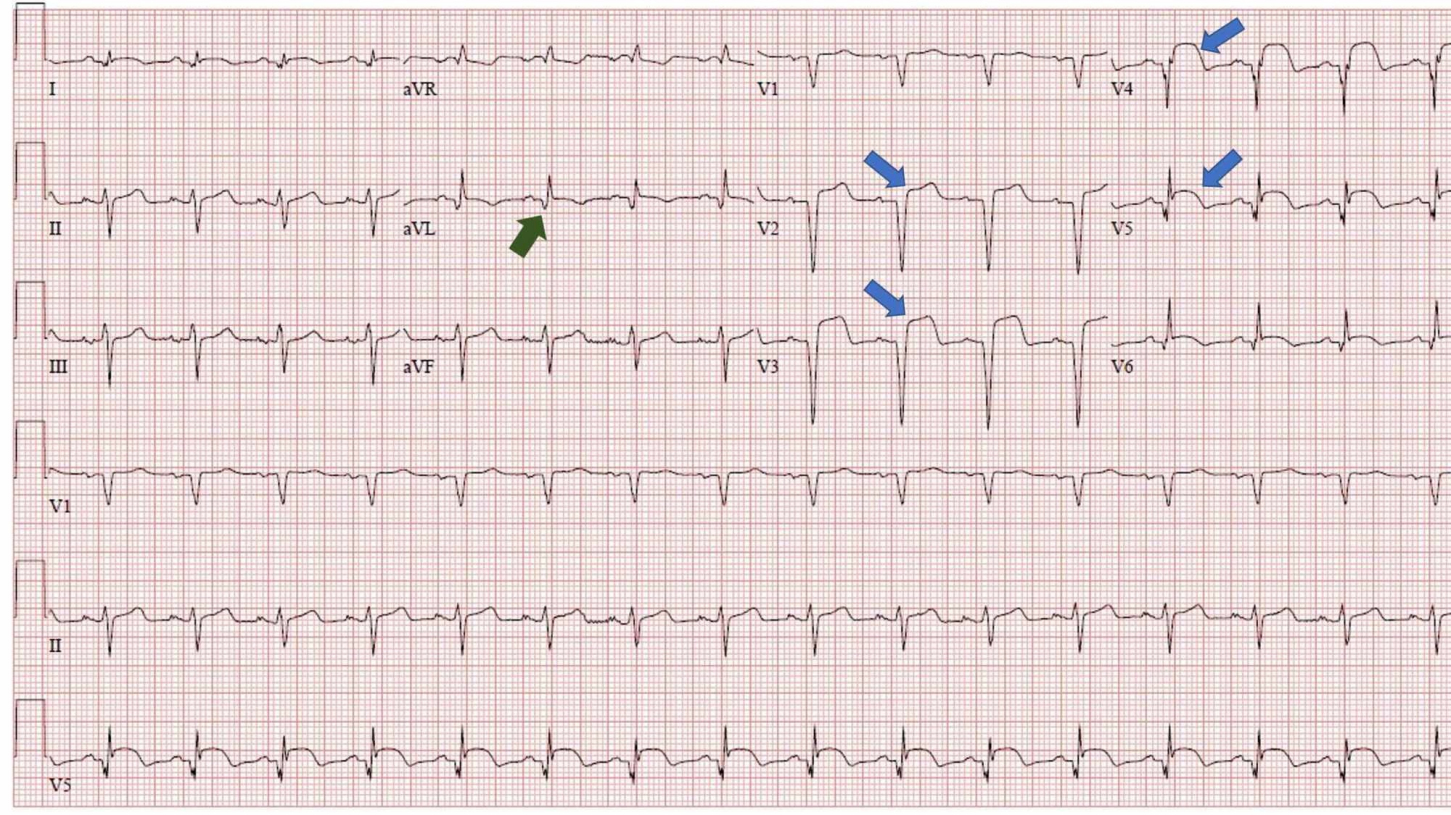
Marked ST-segment elevation in anterior leads and Q wave in aVL 48 h after presentation.

**Figure 7:**
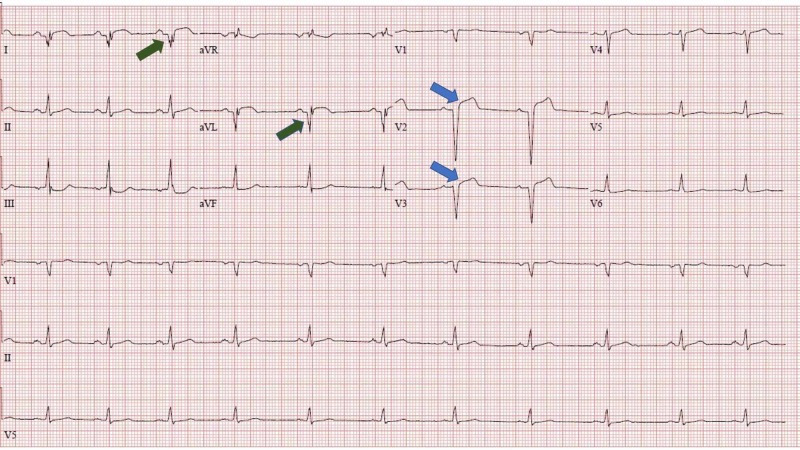
EKG at discharge showing improvement of ST-elevations in anterior leads and Q waves in aVL and I. EKG, electrocardiogram

Echocardiogram showed an ejection fraction of 45% with normal left ventricle (LV) size and mildly impaired LV systolic function. There was also akinesia of the mid/distal anteroseptal, distal lateral, distal septal, distal anterior, distal inferior, and apical walls. Severe hypokinesia of basal anteroseptal was also noticed. There was no LV thrombus or pericardial effusion. The patient was admitted to the cardiac care unit for monitoring. The EKG taken 48 h later showed marked ST elevation V2-V5, and Q waves in aVL (Figure 6) and EKG at discharge demonstrated improvement of acute findings in anterior leads V2-V4 and Q wave progression in aVL and I (Figure 7). The patient was discharged home after four days without any complications.

## Discussion

Acute myocardial infarction (AMI) is a rare but catastrophic complication of BCT. In an extensive review by Christensen et al., he describes 77 cases of BCT causing an MI, of which 64% were related to traffic accidents and 4% were related to a fight [10]. Following a BCT, extensive myocardial contusion may lead to the development of an AMI with typical clinical and ECG findings [11-12].

History, physical examination, EKG, and serial measurement of troponin form the cornerstone of assessment for patients with suspected MI after BCT [13]. The combination of a normal EKG and a normal cTnI (<0.4 ng/mL) almost completely excludes a clinically significant blunt cardiac injury (BCI), with negative predictive values ranging from 98% to 100%. So such patients may be discharged if no other abnormalities are present [14]. Image studies such as plain radiography, ultrasonography, and CT are part of the workup to identify different thoracic injuries such as pneumothorax, parenchymal lung injury, great vessel laceration, esophageal disruption, diaphragmatic laceration, pericardial hemorrhage, among others [15]. In patients with hemodynamic instability or a high suspicion of cardiac tamponade, bedside echocardiogram should be done to quickly identify hemopericardium and to rule out cardiac rupture [16]. A transthoracic echocardiogram can also be helpful to rule out other abnormalities such as a new onset mitral regurgitation, presence of new wall motion abnormalities, or ventricular wall rupture [8].

Our patient initially presented with chest pain from the trauma which was assumed to be musculoskeletal. However, a change in the severity of the pain prompted us to initiate the workup for BCI or a possible MI. Eventually, the patient was found to have a single vessel occlusion with no evidence of atherosclerotic disease in any other artery. Those angiographic findings correlate with other cases in the literature, where intimal tearing leading to intraluminal thrombosis was the most likely cause of AMI [9-10]. The management of ACS associated with BCT is similar to patients without trauma. The ST-segment elevation should prompt immediate coronary angiography with revascularization [17]. Hemodynamically unstable patients with valve, septum, or ventricular wall injury may require emergent surgical intervention [18].

As most patients affected by BCT-induced MI are usually young and have single-vessel disease, the prognosis is relatively favorable [10].

## Conclusions

Acute myocardial infarction after a BCT is a rare but serious complication that can be easily missed. Patients with typical chest pain must be evaluated for a possible MI. EKG and cardiac biomarkers are excellent initial tools and the presence of symptoms should prompt monitoring with serial troponins and EKG every three to six hours. A bedside echocardiogram is a very useful tool to identify life-threatening complications. Patients with STEMI must be evaluated by cardiology immediately for possible emergent intervention. As trauma-induced coronary artery obstruction is the main pathophysiology, coronary angiography is the gold standard for diagnosis and management. As the prognosis of these patients is closely related to early diagnosis and management, it is imperative to keep a high index of suspicion for ACS in patients with BCT and chest pain.
